# *PvARL1* Increases Biomass Yield and Enhances Alkaline Tolerance in Switchgrass (*Panicum virgatum* L.)

**DOI:** 10.3390/plants13050566

**Published:** 2024-02-20

**Authors:** Xue Li, Cong Guan, Huayue Liu, Tingting Wang, Mengzhuo Lin, Die Zhou, Yunwei Zhang, Xiaojing Bi

**Affiliations:** 1 College of Grassland Science and Technology, China Agricultural University, Beijing 100193, China; ml136582@163.com (X.L.); liuhuayue@cau.edu.cn (H.L.); wangtingting@cau.edu.cn (T.W.); linmengzhuo@cau.edu.cn (M.L.); 18701451377@163.com (D.Z.); zywei@cau.edu.cn (Y.Z.); 2 Institute of Leisure Agriculture, Shandong Academy of Agricultural Sciences, Jinan 250100, China; qauguancong@163.com

**Keywords:** switchgrass, *PvARL1*, biomass, alkaline, Na^+^ and K^+^ homeostasis

## Abstract

Switchgrass is an important bioenergy crop valued for its biomass yield and abiotic tolerance. Alkali stress is a major abiotic stress that significantly impedes plant growth and yield due to high salinity and pH; however, the response mechanism of switchgrass to alkali stress remains limited. Here, we characterized *PvARL1*, an ARF-like gene, which was up-regulated in both the shoot and root tissues under alkali stress conditions. Overexpression of *PvARL1* not only improved alkali tolerance but also promoted biomass yield with more tiller and higher plant height in switchgrass. Moreover, *PvARL1* overexpression lines displayed higher capacities in the maintenance of water content and photosynthetic stability compared with the controls under alkali treatments. A significant reduction in the ratio of electrolyte leakage, MDA content, and reactive oxygen species (ROS) showed that PvARL1 plays a positive role in protecting cell membrane integrity. In addition, PvARL1 also negatively affected the K^+^ efflux or uptake in roots to alleviate ion toxicity under alkali treatments. Overall, our results suggest that PvARL1 functions as a positive regulator in plant growth as well as in the plant response to alkali stress, which could be used to improve switchgrass biomass yield and alkali tolerance genetically.

## 1. Introduction

Saline–alkaline stress inhibits plant growth. Over 1 billion hectares of soil were found to be affected by salt, of which 40% and 60% were classified as saline and sodic soils, respectively, thus seriously threatening human and animal food supplies [1]. Different from saline soil, which mainly contains sodium chloride (NaCl) and sodium sulfate (Na_2_SO_4_) or other neutral salts, sodic soils usually over-accumulate sodium bicarbonate (NaHCO_3_) and sodium carbonate (Na_2_CO_3_), leading to alkaline soils with a pH of over 8.2 [2]. Therefore, plants undergo a higher risk under alkaline stress compared with salinity stress by neutral salts [3]. However, alkali stress on plants has yet to raise considerable research attention [1]. Improving the alkali tolerance of plants is crucial to maintaining environmental sustainability. Due to the importance of improving plant alkali tolerance for increasing food demands and protecting the environment, it is essential to understand alkaline response mechanisms in plants. 

Previous studies have shown that many genes could be simultaneously involved in salt and alkali stress in various plant species, like *Miscanthus sinensis*, soybean, and grapevine [4,5,6]. *Soybean GmSNF1 (SUCROSE NON-FERMENTING PROTEIN KINASE 1)* was up-regulated and *Miscanthus sinensis MsGRAS60* (GAI (gibberellic acid-insensitive), RGA (a repressor of GAI), and SCR (scarecrow)) was down-regulated under salt and alkali stress [5,6]. Ectopic-expressing *Glycine max GmPKS4* (*CALCINEURIN B-LIKE PROTEIN-INTERACTING KINASES 4*) in Arabidopsis and *Medicago sativa MsCBL4* (CALCINEURIN B-LIKE 4) in tobacco both enhanced salt and alkali tolerance of the corresponding plants [7,8]. The overexpression of *Osa-MIR393* in rice and Arabidopsis resulted in more sensitivity to salt and alkali stress [9]. However, studies have shown that the detrimental effects of alkali stress on the growth and photosynthesis of wheat were more severe than those caused by salt stress [10]. Apart from stress-specific responses, a considerable number of genes respond to both salt and alkali stress, allowing for the identification of alkali-tolerant genes based on known salt-tolerant ones, thus expediting the breeding of alkali-tolerant varieties.

The ADP-ribosylation factor (ARF) family of small GTP-binding proteins belongs to the Ras superfamily, which was originally identified due to inactivating the cholera toxin [11]. ARF proteins were then found to function in multiple aspects, including the regulation of various intracellular signaling and vesicular trafficking pathways and the abiotic stress response in eukaryotic cells [12,13,14]. The ARF family proteins are classified into two groups based on their sequence homology and functional characteristics: ARF and ARF-like (ARL) proteins [15]. ARFs are highly conserved proteins (>60% sequence identity) sharing similar biological activities, but ARLs are more divergent (40–60% sequence identity) and function in secretory and other pathways [16]. The Longan *DlARL1*, *DlARL2*, and *DlARL8a* promoter sequences were found to contain abiotic stress response elements [17]. The ectopic expression of *Medicago falcate MfARL1* in Arabidopsis enhanced the salt tolerance of the transgenic plant [18]. Previous research suggested that the overexpression of *PvARFs* (*PvArf1*-, *PvArf-B1C*-, and *PvArf-related*) in switchgrass resulted in improved plant growth and enhanced salt tolerance [19]. However, there has been no research on the response of *ARL* genes to alkali stress in switchgrass so far. Given the conserved sequences between ARLs and ARFs, we hypothesize that *PvARLs* might play a role in the alkali stress response in switchgrass. 

Therefore, we used the *PvARL1*-overexpressed and *PvARL1*-RNAi lines, to reveal its function and regulatory mechanism in switchgrass under alkali stress, aiming to gain a theoretical basis and targeted gene resources for alkali-tolerant crop breeding. 

## 2. Materials and Methods

### 2.1. Plant Materials and Growth Conditions

The seeds of the cultivar “Alamo”, a lowland switchgrass ecotype, were immersed in 5% NaClO (*v*/*v*) for 1 h with shaking every 15 min, followed by washing five times with sterilized water and germinating on wet filter paper in growth chambers at 25 °C and a 16 h light/8 h dark photoperiod for 7 days. The seedlings were cultured in a hydroponic system with 1/4 Hoagland solution until the three-leaf stage and were then used for detecting *PvARL1* expression patterns at different time points under alkali stress. 

All the transgenic plants were generated from the callus originating from the same Alamo seed, according to the switchgrass transformation protocol described previously [20]. The plants with empty vectors (EVs) and non-transgenic (NT) plants were used as controls for the overexpressed and RNAi lines, respectively. After producing enough tillers, the transgenic and control lines were propagated by separated tillers. Each tiller was trimmed to both shoots and roots of 5 cm in length before replanting in pots filled with pure vermiculite to ensure the consistency of each replicate in development. The plants, including the EV, *PvARL1-*OE lines and the NT, *PvARL1-*RNAi lines, were cultured in a greenhouse with natural light at a temperature ranging from 30 °C to 25 °C during the day/night. 

The phenotypes EV, *PvARL1*-OE and NT, *PvARL1*-RNAi were measured at the R3 stage and three independent transgenic lines. 

### 2.2. Plasmid Construction and Plant Transformation in Switchgrass

Full-length coding sequences of *PvARL1* were cloned from the switchgrass ecotype Alamo. Corrected sequences were introduced into the binary vector Ubi1301 by restriction enzymes *BamH*I/*Kpn*I (New England BioLabs, Ipswich, MA, USA). A 200 bp distinctive fragment of *PvARL1* was selected for RNAi via generating a hairpin structure in the pEntry/D-Kannibal and then recombined into the destination vector pVT1629 by the LR reaction, as indicated in the user manual (Invitrogen, Carlsbad, CA, USA, Gateway LR Clonase).

The constructed vectors were introduced into EHA105 *Agrobacterium tumefaciens* by freeze–thaw transformation. The control plants for the *PvARL1-OE* lines were generated with transformations of empty vectors (EVs) of Ubi1301 into switchgrass, while the controls for the *PvARL1-RNAi* lines were the non-transgenic (NT) plants. All primers used in this study are listed in Supplemental Table S1. 

### 2.3. Abiotic Tolerance Assay in Switchgrass

The three-leaf stage seedlings, cultured hydroponically as mentioned in Section 2.1, were treated with 0 or 150 mM Na^+^, NaHCO_3_:Na_2_CO_3_ = 9:1 alkaline salt in 1/4 Hoagland solution for 0 h, 3 h, 6 h, 12 h, 24 h, and 48 h. The shoots and roots were split and, respectively, collected from three replicates of each genotype for detecting the relative expression of *PvARL1* by RT-qPCR. 

We tested the alkaline tolerance of switchgrass transgenic plants in a vermiculite-culture system using the transgenic and control plants mentioned in Section 2.1. Until most of the newly generated shoots from the trimmed tillers reached the developmental stage of E3, i.e., the elongation 3 stage [21], 1/4 Hoagland solution containing 0 or 150 mM Na^+^ (NaHCO_3_:Na_2_CO_3_ = 9:1, pH 9.0) was applied every third day to the plants. To avoid stress shock, a solution with 50 mM Na^+^ was watered for the first time, followed by solutions with 100 mM and 150 mM Na^+^. The PvARL1-OE and EV lines were exposed to alkali stress for 14 days, and then leaf and root samples from each genotype were used to determine physiological and chemical changes under alkali stress. Three independent replicates of each genotype were used.

### 2.4. RNA Extraction and RT-qPCR

The total RNA of plants was isolated using a Beijing Hua Yueyang Kit, and 1 μg of total RNA was used for cDNA reverse transcription using a PrimeScript™ RT reagent Kit with gDNA Eraser (Takara, RR047A). qPCR was performed using SYBR Green supermix (Takara, RR420A) and qTOWER^3^ G (Analytik Jena, Jena, Germany). Transcript levels were normalized to the control *PvUbiquitin1* (AP13CTG25905) [22] in switchgrass.

### 2.5. Na^+^ and K^+^ Content Measurements

The leaves and roots were harvested and dehydrated at 60 °C for 3 days. Then, all the samples were shaken well to suspend in the 5% HNO_3_ for 2 days at room temperature. The K^+^ and Na^+^ contents were measured by a 4100-MP-AES device (Agilent, Santa Clara, CA, USA) as previously reported [23]. 

### 2.6. Chlorophyll Content and Fv/Fm Measurements

The leaves were soaked in 95% ethyl alcohol for 48 h in the dark, and the extract was measured at 665 nm, 649 nm, and 470 nm using a spectrophotometer (PuXi General instrument, T6 New Century, Beijing, China) for chlorophyll content. 

The leaves were adapted to the dark for 30 min, and then the *Fv*/*Fm* values were measured by a fluorescence meter (Opti-sciences, OS30p+, Beijing, China). 

### 2.7. Relative Water Content and Electrolyte Leakage Measurements

The relative water content and electrolyte leakage of the leaves were performed as previously described [24]. The leaves were soaked in deionized water and left to absorb water to saturation, and then their weight was measured. Then, the leaves were dehydrated at 65 °C for 24 h, and then their weight was measured again and the relative water content was calculated. For electrolyte leakage, the leaves were soaked in deionized water and shaken for 24 h followed by autoclave sterilization for 30 min and shaking for 24 h, and then the electrolyte leakage was measured. 

### 2.8. MDA and H_2_O_2_ Accumulation Analysis

The H_2_O_2_ content was measured by a H_2_O_2_ kit as indicated in the user manual (COMIN, H_2_O_2_-1-Y). Briefly, 0.1 g of the control and alkali-stressed switchgrass leaves were harvested and homogenized in 1 mL of prechilled acetone, and then the H_2_O_2_ content was measured. 

To test MDA content, 0.1 g of the control and alkali-stressed switchgrass leaves were harvested and measured by an MDA kit (COMIN, MDA-1-Y) according to the user manual. 

### 2.9. Subcellular Localization

CDS without the stop codon of *PvARL1* was cloned into the *pMDC83*-GFP vector by the gateway method to generate a GFP fusion construct. It was then introduced into the GV2260 *Agrobacterium* strain and co-infiltrated together with the P19 suppressor and marker of the cell nucleus into 4-week-old *N. benthamiana* leaves and co-cultivated for 48 h to 60 h. The GFP signal (excitation at 488 nm and scanning at 561 nm) was observed by confocal laser scanning microscopy (Nikon, A1, Tokyo, Japan).

### 2.10. Statistical Analysis

The Duncan’s test (*p* ≤ 0.05) was used for pairwise multiple comparisons. The data analysis was performed with GraphPad Prism version 8 and SPSS 23 software. The error bars in RT-qPCR and physiological parameter figures show the standard deviation of three biological or technical replicates, as indicated in the legends. 

## 3. Results

### 3.1. PvARL1 Responses to Alkaline Stress

**Figure 1:**
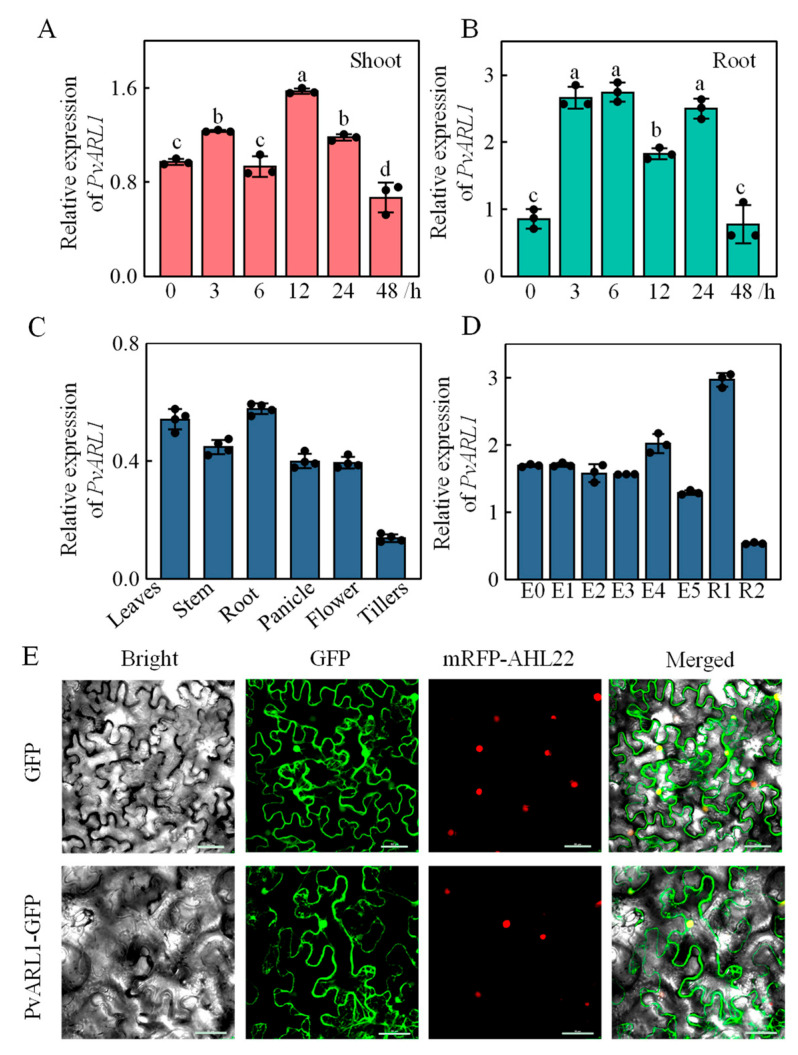
Expression patterns of *PvARL1* and subcellular localization. (**A**,**B**): Expression levels of *PvARL1* transcripts in shoots (**A**) and roots (**B**) under a series time-course of alkali treatment. Fourteen-day-old “Alamo” seedlings were transferred to 1/4 Hoagland solution with or without 150 mM Na+, NaHCO_3_:Na_2_CO_3_ = 9:1 from 3 h to 48 h. Data are the means of three biological replicates (±SD). Different letters represent statistically significant differences as indicated by Duncan analysis by SPSS 23 software. (**C**,**D**): Expression patterns of PvARL1 in different tissues (**C**) and leaves at different development stages (**D**). E0–E5, the youngest leaf of plants at the 5-elongation vegetative stage; R1–R2 indicate the flag leaf at different reproductive stages (Hardin et al., 2013) [21]. (**E**): Subcellular localization of PvARL1. Nuclear protein AHL22 was used as a nuclear localization marker. Bars = 50 µm.

To explore whether the *PvARL1* gene responds to alkaline stress, three-leaf switchgrass seedlings were treated under alkali conditions from a two-day time course. The shoots and roots were harvested, respectively, at 0 h, 3 h, 6 h, 12 h, 24 h, and 48 h for transcripts detection. The RT-qPCR analysis results showed that transcripts of *PvARL1* in the shoots were upregulated at 3 h and then peaked at 12 h with a 1.6-fold increase compared with the nontreatment (Figure 1A). The expression of *PvARL1* in the roots under alkaline stress was also induced, which was significantly elevated after 3 h and reached a peak after 6 h with about 2.7 folds of the expression in the control (Figure 1B). The expression levels of *PvARL1* in both shoots and roots were weaker after the peak and then disappeared after 24 h of alkaline treatment (Figure 1A,B). In summary, *PvARL1* could respond fast in both shoots and roots under alkaline stress, indicating that *PvARL1* is involved in the alkaline response of switchgrass. 

### 3.2. Expression Pattern and Subcellular Location of PvARL1

To investigate the expression pattern of *PvARL1*, tissues at different developmental stages were collected for transcriptional detection. The RT-qPCR results showed that *PvARL1* was expressed in all tested tissues, including the leaves, stems, roots, panicles, flowers, and tillers (Figure 1C). Moreover, *PvARL1* was expressed not only in the vegetative stages (E0, E1, E2, E3, E4, and E5) [21] but also in the reproductive stages (R1 and R2), with the highest expression level at the R1 stage (Figure 1D), the early reproductive stage, indicating that *PvARL1* might respond to alkaline stress at both vegetative and reproductive stages and have a stronger response at early reproductive stage. 

To determine the subcellular localization of PvARL1, we constructed PvARL1-fused GFP at the C-terminus driven by the 35S promoter. The corrected construct was infiltrated with a nucleus localized marker mRFP-AHL22 into *N. benthamiana* leaves for fluorescent detection. Similar to the GFP control, the PvARL1-GFP fusion protein localized to the cytoplasm and nucleus (Figure 1E).

### 3.3. PvARL1 Positively Regulates Plant Growth

**Figure 2:**
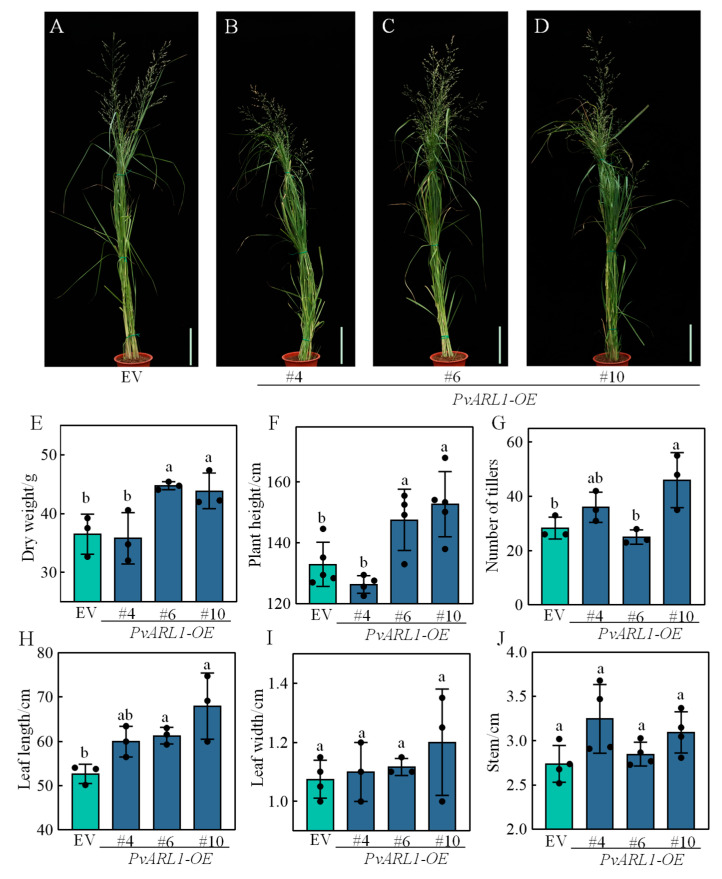
Phenotypes of overexpressing *PvARL1* transgenic switchgrass lines. (**A**–**D**): Phenotypes of the R3-stage empty vector (EV) and three independent transgenic lines overexpressing *PvARL1*. Bars = 15 cm. (**E**–**J**): Total aboveground dry weight of the R3 stage (**E**), plant height of the R3 stage (**F**), tillers (**G**), length of the third leaf from the top (**H**), width of the third leaf (**I**), and stem diameter of the second internode from the base (**J**). Different letters represent statistically significant differences as indicated by Duncan’s test calculated by SPSS 23 software.

To explore the function of *PvARL1*, we constructed *PvARL1* driven by the Ubiquitin promoter to generate overexpression lines (*PvARL1*-OE*)*. A total of 12 independent lines were obtained with higher transcripts compared with the control, and 3 out of 12 lines were used for further study (Figure S1A). We first examined the growth traits related to the biomass yield. Two independent *PvARL1*-OE lines #6 and #10 showed a significant increase in plant height and dry biomass (Figure 2A–F) and produced significantly longer leaves, while no significant changes or clear trends were found in the leaf width (Figure 2H,I). Except for plant height and biomass, only overexpression line #6 exhibited significantly more tillers compared with the controls (Figure 2G). In addition to the significant changes according to statistics, it showed a clear trend of an increase in stem diameters of the *PvARL1*-OE lines (Figure 2J).

**Figure 3:**
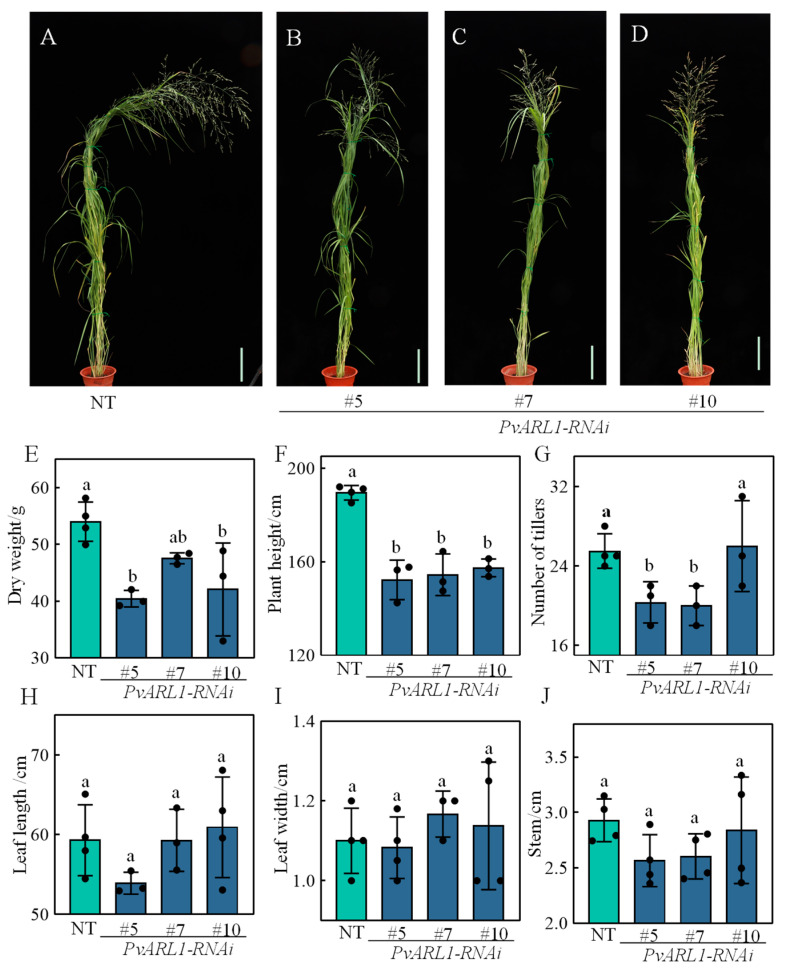
Phenotype and biomass analysis of switchgrass *PvARL1-RNAi* lines. (**A**–**D**): Phenotypes of the R3 stage non-transgenic (NT) and three independent transgenic lines *PvARL1-RNAi*. (**E**–**J**): Total aboveground dry weight of the R3 stage (**E**), plant height of the R3 stage (**F**), tillers (**G**), length of the third leaf from the top (**H**), width of the third leaf (**I**), and stem diameter of the second internode from the base (**J**). Different letters represent statistically significant differences as indicated by Duncan’s test calculated by SPSS 23 software.

*PvARL1* RNA-interfered (*PvARL1*-RNAi) switchgrass lines were also generated for further study. Nine independent transformants with downregulation transcripts were obtained (Figure S1B), and three out of nine *PvARL1*-RNAi lines (#5, #7, #10) were selected for phenotyping compared with a non-transformant. Under normal growth conditions, the plant height and biomass were significantly shorter and less compared with the NT control (Figure 3A–F). Also, the tillers of *PvARL1*-RNAi #5 and #7 exhibited fewer than the control tillers (Figure 3G). Additionally, no significant changes or clear trends were found in leaf length or width (Figure 3H,I). The stem diameters showed a reduced trend in *PvARL1*-RNAi, although no significant changes occurred according to the statistics (Figure 3J). Taken together, the plant height and dry weight in the *PvARL1*-OE lines were significantly higher but were lower in RNAi plants than in each control, indicating that *PvARL1* likely promoted switchgrass growth and development by increasing plant height, leading to higher biomass. 

### 3.4. PvARL1 Alleviates Leaf Damage under Alkali Conditions

**Figure 4:**
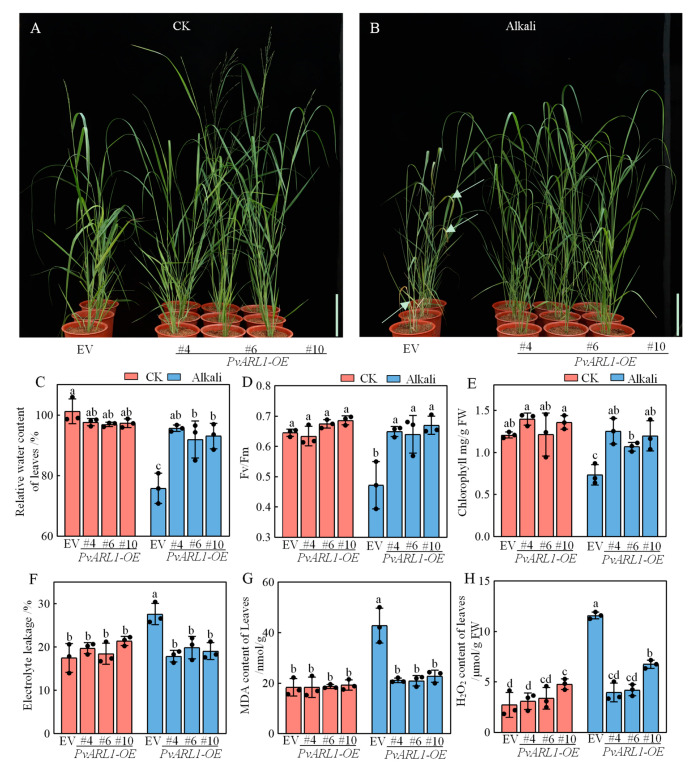
Overexpression *PvARL1* improves switchgrass alkaline tolerance. (**A**,**B**): Phenotype analysis of *PvARL1* overexpression lines under normal conditions in (**A**) (0 mM Na^+^ with 1/4 Hoagland solution, pH = 7) and alkaline stress in (**B**) (150 mM Na^+^ with 1/4 Hoagland solution, NaHCO_3_:Na_2_CO_3_ = 9:1, pH = 9). E3-stage plants were exposed to normal or alkaline solution for another 14 days. Bars = 15 cm. (**C**–**H**): Relative leaf content (**C**), *Fv*/*Fm* (**D**), chlorophyll content (**E**), electrolyte leakage (**F**), MDA content (**G**), and H_2_O_2_ content (**H**) in EV and *PvARL1*-OE under normal and alkaline condition for 14 days. Different letters represent statistically significant differences as indicated by Duncan’s test calculated by SPSS 23 software.

The quick response of *PvARL1* to alkali in Figure 1 led us to investigate the phenotypes of transformants in detail under alkali treatment. Firstly, we conducted a detached leaf assay under alkali stress using the leaves from the *PvARL1*-OE and EV lines at the vegetative stage of E3. In contrast to the control treatment, the three *PvARL1*-OE lines all exhibited smaller yellowing areas of treated leaves compared with those of the EVs under alkali stress (Figure S2), suggesting that *PvARL1* improved alkali stress tolerance in switchgrass. Next, we further investigated the function of *PvARL1* in response to alkali in soil conditions. The EV plants as a control and the *PvARL1*-OE lines were cultured in soil with or without 150 mM Na^+^, NaHCO_3_:Na_2_CO_3_ = 9:1 for 14 days. Under control growth conditions, the EV and *PvARL1*-OE plants grew well and stayed green, although the *PvARL1*-OE lines displayed a faster growth rate than the EV ones (Figure 4A). After alkali treatment, the difference in plant height between the EV control and the *PvARL1*-OE lines was more visible. Leaves of EV control plants exhibited visible withered and yellowish leaves compared with the overexpressed lines, indicating that the EV lines suffered from alkali stress more severely than the *PvARL1*-OE lines (Figure 4B). One typical phenomenon for plant sensitivity to alkaline environments is leaf damage, which is always indexed by the water loss ratio and chlorophyll. Consistent with the appearance, the three *PvARL1*-OE lines all exhibited higher relative water rates compared with the EV control under alkaline stress (Figure 4C); similarly, *Fv*/*Fm* as well as the chlorophyll contents also displayed higher in the *PvARL1*-OE lines (Figure 4D,E). 

### 3.5. PvARL1 Overexpression Decreases Oxidative Damage after Alkali Treatment

It is well known that abiotic stresses destroy cell membrane integrity, indicated by electrolyte leakage. No differences existed in electrolyte leakage in leaves between the EV and *PvARL1*-OE transgenic plants under control growth conditions. In contrast, electrolyte leakage was significantly lower in all three *PvARL1*-OE overexpressed lines than in EV plants after alkali treatment (Figure 4F).

To further investigate whether the overexpression of *PvARL1* would decrease oxidative damage under alkali conditions, the quantification of H_2_O_2_ content was tested. The accumulation of H_2_O_2_ in all three *PvARL1*-OE leaves was lower than the EV under alkali treatment, whereas no differences under control growth conditions were observed (Figure 4H). In addition, malondialdehyde (MDA) concentration is a valid indicator of cytomembrane oxidative damage in monitoring ROS levels. Under normal conditions, there were no differences in MDA content between the *PvARL1*-OE and EV transformants. However, the MDA content in the *PvARL1*-OE lines was lower than that in the EV plants after alkali stress (Figure 4G). In addition, expression levels of the ROS scavengers *PvSOD* and *PvCAT* were measured. Under normal conditions, the qPCR analysis showed no differences between the EV and *PvARL1*-OE lines, whereas the transcripts of *PvSOD* and *PvCAT1* accumulated in the *PvARL1* lines under alkali stress conditions (Figure S3). These results suggest that *PvARL1* promoted alkaline stress tolerance better because of the capacity to maintain cell membrane stability and accumulate fewer ROS under alkali stress.

### 3.6. PvARL1 Maintained Ion Homeostasis in Switchgrass

**Figure 5:**
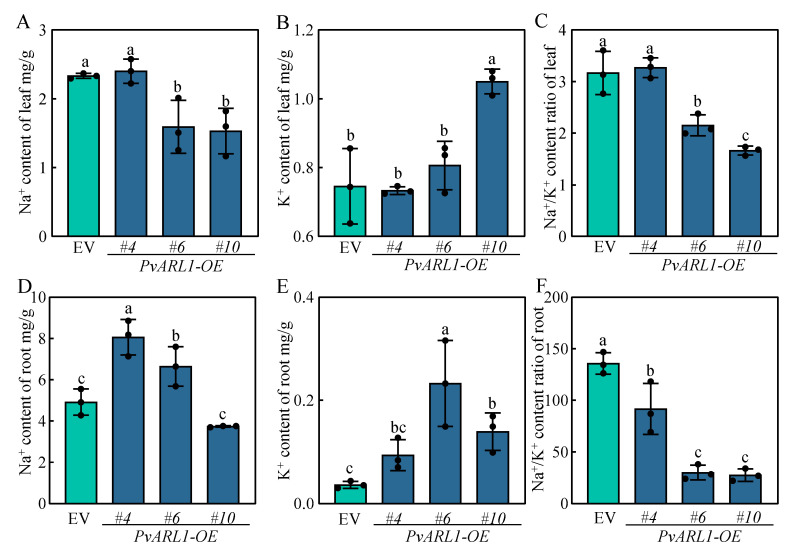
Na^+^ and K^+^ uptake of transgenic lines under alkaline stress/under control conditions. (**A**–**C**): Na^+^ content (**A**), K^+^ content (**B**), Na^+^/K^+^ ratio (**C**) in leaves after 14 days under alkaline stress/control conditions. (**D**–**F**): Na^+^ content (**D**), K^+^ content (**E**), and the Na^+^/K^+^ ratio (**F**) in roots after 14 days under alkaline stress/control conditions. Different letters represent statistically significant differences as indicated by Duncan’s test calculated by SPSS 23 software.

A low cytosolic Na^+^/K^+^ ratio in the cytoplasm is necessary for plants to maintain iron homeostasis under salt, salt–alkali, and alkali stresses. To investigate whether *PvARL1* plays a role in this process under alkaline stress in switchgrass, we measured Na^+^ and K^+^ content and calculated the Na^+^/K^+^ ratio in the roots and leaves separately under normal and alkaline conditions. Consistent with the phenotypes, the *PvARL1* overexpression lines were more resistant to alkaline stress than the control as they displayed significantly lower Na^+^ content in their leaves, a higher K^+^ content in their roots, and a lower Na^+^/K^+^ ratio in both their leaves and roots under alkaline stress (Figure 5). We next tested the expression levels of the High-Affinity K^+^ Transporter *PvHAK5* in the *PvARL1*-OE lines by RT-qPCR. Transcripts of *PvHAK5* were significantly higher compared with EV, whereas no difference between the EV and *PvARL-*OE lines were observed (Figure S4). Those results demonstrated that *PvARL1* played a positive role in alkaline tolerance by regulating Na^+^ and K^+^ homeostasis under alkaline treatments. 

## 4. Discussion

Small G proteins including Ras-like in the brain (Rab), Rho in plants (Rop), ADP-ribosylation factor (Arf), and Ras-like nuclear (Ran) have been identified and demonstrated to play a role in plant growth and the abiotic stress response [25,26,27]. In *Arabidopsis thaliana*, the overexpression of a Ran family gene *AtRAN1* promoted transgenic plants’ vegetative growth and enhanced their tolerance to salt, ABA, and freezing, whereas the *atran1 atran3* double mutant exhibited opposite phenotypes [28,29]. Additionally, ectopically expressing maize *ZmARF2* in Arabidopsis increased leaf and seed size, thus augmenting overall plant growth [30]. Overexpressing *PvARF1*, *PvARF-B1C*, and *PvARF-related* in switchgrass enhanced biomass by improving tillers, height, and leaf length [19]. ARF family proteins are classified into two subclades: ARFs and ARLs. ARLs have been extensively studied in mammals compared with plants, leaving a significant gap in our understanding of the gene functions in plants. Similar to ARFs, we found that the overexpression of *PvARL1* increased plant height and dry weight, indicating the conserved roles of small G protein family members during plant growth and development.

In this study, *PvARL1* was significantly induced in both shoots and roots under alkali stress (Figure 1), and the *PvARL1* overexpression lines showed promoted growth in plant height and biomass compared with the control under normal or alkali conditions (Figure 4A,B). These results strongly suggest that alkali-induced *PvARL1* plays a critical role in alkali tolerance. In addition, the results in this study mirror the behavior of *OsARL1a*, which shows upregulation under NaCl, PEG, and ABA stress conditions [31]. However, *OsARL1b*, *OsARL1d,* and *OsARL1e* did not exhibit any changes under abiotic stress conditions [31], indicating diverse functionalities among *ARL* genes in plants. 

One of the key physiological aspects affected by various abiotic stresses, including salt, alkali, and drought, is the relative water content and photosynthetic capacity in plants. Chlorophyll is an important pigment in photosynthesis, reflecting plants’ ability to assimilate substances [32,33]. *Fv*/*Fm* is an index of the maximum photochemical efficiency of PSII in the dark-adapted state, and a decrease in this parameter is a reliable sign of photoinhibition [34]. Here, we found that the relative water content in the leaves of the *PvARL1*-OE lines was significantly higher than that in the EV lines under alkali stress (Figure 4C). Under alkali stress conditions, *PvARL1*-OE switchgrass exhibited higher chlorophyll levels and a greater *Fv*/*Fm* ratio compared with the EV plants (Figure 4D,E). This suggests that *PvARL1* mitigates the adverse effects of alkali stress on the chlorophyll content and photoinhibition, thereby preserving the higher photosynthetic capacity in the *PvARL1*-OE lines. 

Excessive accumulation of reactive oxygen species (ROS) produced by abiotic stresses leads to oxidative conditions that can disrupt cellular homeostasis, evidenced by increased levels of H_2_O_2_ and MDA [35]. It is noteworthy that small GTPases regulate plant responses to abiotic stresses through pathways involving reactive oxygen species, as reported in numerous other plant species. The heterologous expression of *Medicago falcata MfARL1* in Arabidopsis enhanced salt tolerance by elevating CAT (catalase) activities and reducing the accumulation of H_2_O_2_ and MDA compared with the wild-type under salt stress [18]. In switchgrass, overexpressing *PvARF* genes enhanced salt tolerance, and the H_2_O_2_ content was significantly lower compared with that of the WT, indicating a reduction in oxidative damage caused by stress [19]. Excess production of ROS exerts toxic effects on plant cell metabolism. Nevertheless, plants have evolved multiple antioxidant systems to tightly regulate an appropriate amount of ROS [36]. It has been proven that the overexpression of key antioxidant genes, such as SUPEROXIDE DISMUTASES (SOD) and CATALASE (CAT), effectively improves plant abiotic stress tolerance by increasing ROS scavenging capacity in a variety of plant species [37,38]. In this study, the control plants accumulated higher levels of H_2_O_2_ and MDA when exposed to alkali stress (Figure 4G,H). However, the *PvARL1*-OE transgenic switchgrass exhibited a lower accumulation of H_2_O_2_ and MDA content under alkali stress conditions, even equal to the control growth conditions (Figure 4G,H). The ROS-scavenging enzyme genes *PvSOD* and *PvCAT* were significantly upregulated in the *PvARL1*-OE lines compared with that of EVs (Figure S3). These results indicate that the promoted alkali tolerance of the *PvARL1* overexpression plants was attributed to their lower ROS content levels. 

Additionally, the *PvARL1* overexpression lines displayed higher Na^+^ content in their roots compared with the EVs, yet in the leaves, the Na^+^ content was significantly lower in the overexpression lines than in the EVs. Those results imply that Na^+^ could accumulate in the roots, yet the transportation to the leaves is reduced, thus mitigating ion toxicity. Meanwhile, the *PvARL1* overexpression lines maintained a higher level of K^+^ in their roots compared with the EVs, but there was no clear trend in K^+^ content in the leaves. The *PvHAK5* expression level in the *PvARL1*-OE lines was upregulated compared with the EV control under alkali conditions, which has been reported to enhance K^+^ uptake in a lower K^+^ environment [39]. These results imply that the *PvARL1* maintenance of a higher level of K^+^ content in the roots might be attributed to reduced K^+^ efflux or maintaining a higher selective absorption of K^+^ under alkali stress, thus playing a crucial role in preserving ion homeostasis. It has been reported that electrolyte leakage primarily corresponds to the efflux of K^+^, mediated by plasma membrane cation conductance [40]. This aligns with the findings of our study, wherein the *PvARL1*-OE lines exhibited lower electrolyte leakage compared with the EVs under alkali stress. Collectively, these results highlight the involvement of *PvARL1* in plant growth and illustrate how the overexpression of *PvARL1* enhances alkali tolerance in switchgrass. 

## 5. Conclusions

In this study, *PvARL1* exhibited induction in both shoot and root tissues in response to alkali stress, and the overexpression of *PvARL1* notably enhanced alkali tolerance in switchgrass. Physiologically, the improved alkali tolerance observed in *PvARL1*-overexpressing lines can be attributed to the maintenance of water content and photosynthetic stability in switchgrass. Additionally, these lines showed a reduced accumulation of reactive oxygen species (ROS), maintained cell membrane integrity, and exhibited decreased K^+^ efflux or uptake in the roots, potentially alleviating ion toxicity under alkali stress. Our research also revealed that the *PvARL1* gene contributes to enhanced plant growth and increased biomass yield under normal conditions.

Moving forward, our future investigations will delve deeper into uncovering the regulatory mechanisms of *PvARL1* in switchgrass under alkali stress. Understanding the mechanisms holds significant promise for utilizing switchgrass as a valuable resource in salt–alkali soil applications. 

## Data Availability

The data presented in this study are available in the figures and tables provided in this manuscript.

## Supplementary Materials

The following supporting information can be downloaded at: https://www.mdpi.com/article/10.3390/plants13050566/s1, Figure S1: Relative expression of PvARL1 in PvARL1-OE and PvARL1-RNAi transgenic switchgrass. Figure S2: Phenotypes analysis of detached leaves of PvARL1-OE under alkali stress. Figure S3: Relative expression of PvSOD (A) and PvCAT (B) in PvARL1-OE transgenic switchgrass. Figure S4. Relative expression of PvHAK5 in PvARL1-OE transgenic switchgrass. Table S1: Primers used in this study.
