# Supplementary material for: PvARL1 Increases Biomass Yield and Enhances Alkaline Tolerance in Switchgrass (Panicum virgatum L.)
Source: Plants (Basel). 2024 Feb 20;13(5):566. doi: 10.3390/plants13050566 (PMC10934731; doi:10.3390/plants13050566)
Supplement: Supplementary file 1 [file plants-13-00566-s001.zip › plants-2833765-Supplement Table S1.pdf]

Supplement Table S1 Primers used in this study

Primers	Sequence (5'-3')
PvARL1-F (qRT)	AGGTGCTCTCGATGATGCTG
PvARL1-R (qRT)	GAGCCAGTCCAAACCTTCGA
PvARL1-attB1-F	ggggacaagtttgtacaaaaaagcaggcttcATGGGCATCGTCTTCAC
PvARL1-attB2-NS-R	ggggaccactttgtacaagaaagctgggtcGCTGCTTCCGGATTTGAGT
PvARL1-F	ATGGGCATCGTCTTCAC
PvARL1-R	CTAAATTTGTCCAGCTACAC
PvARL1-RNAi-F	TCTAGAGTCGACTTCTCCCTGTTCTATGGCCT
PvARL1-RNAi-R	AAGCTT GAATTCCATCTTCAATGCGGCCAATG
PvHAK5-F (qRT)	TGGTGATGGCGTTCTAACCC
PvHAK5-R (qRT)	ACCCAACTTTGTCTGTGCCA
PvSOD-F (qRT)	CTGGACCGCATTACAACCCT
PvSOD-R (qRT)	TGAGTTTGGCCCAGTCAGTG
PvCAT-F (qRT)	TTCCTATCCCTCCCCGTGTT
PvCAT-R (qRT)	TCTCACCAGCCTGCTTGAAG
