# Supplementary material for: PvARL1 Increases Biomass Yield and Enhances Alkaline Tolerance in Switchgrass (Panicum virgatum L.)
Source: Plants (Basel). 2024 Feb 20;13(5):566. doi: 10.3390/plants13050566 (PMC10934731; doi:10.3390/plants13050566)
Supplement: Supplementary file 1 [file plants-13-00566-s001.zip › plants-2833765-supplementary.pptx]

## Slide 1
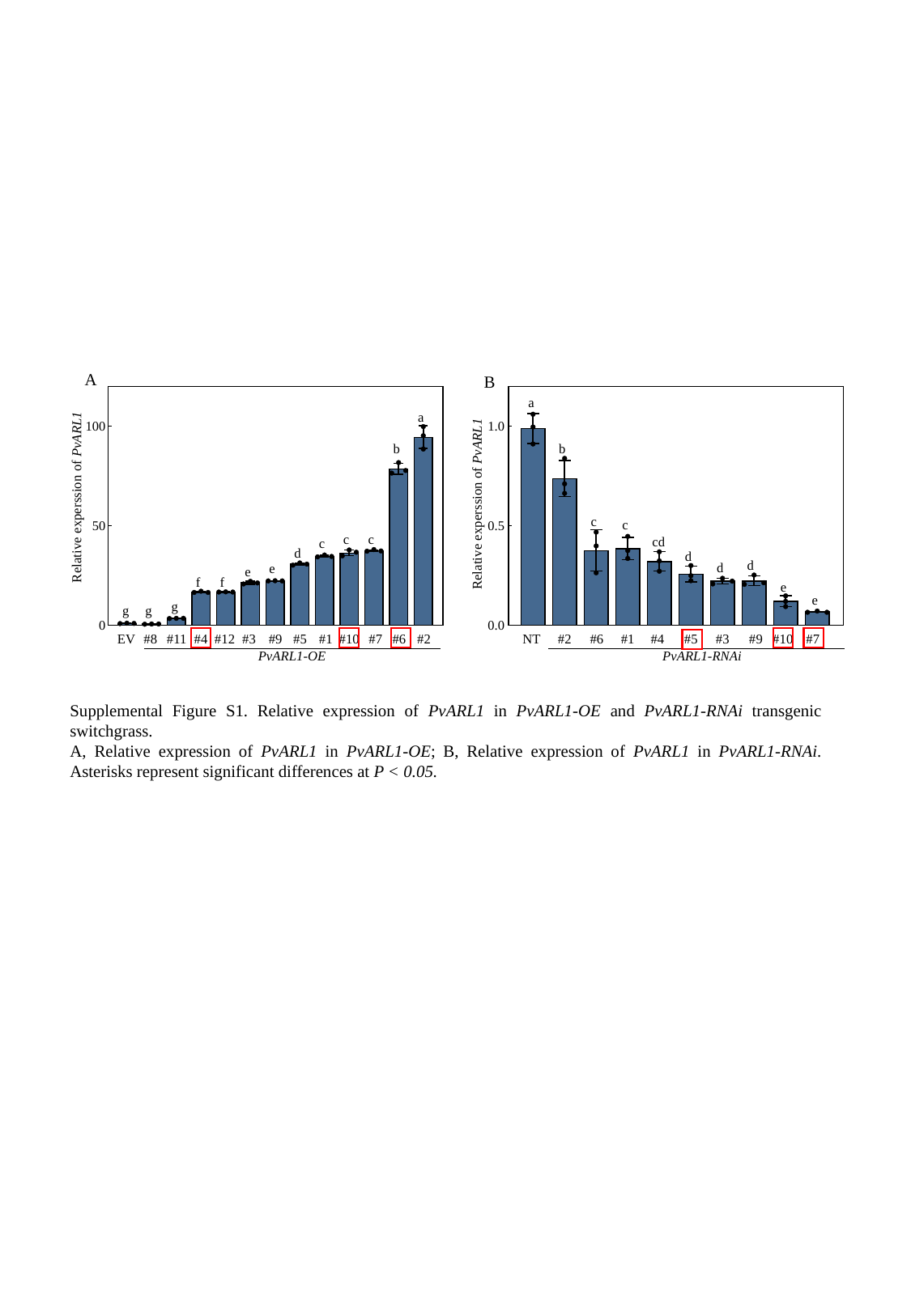

a
b
Relative experssion of PvARL1
c
c
c
d
e
e
f
f
g
EV
#8
#11
#4
#12
#3
#9
#5
#1
#10
#7
#6
#2
PvARL1-OE
Relative experssion of PvARL1
c
c
cd
d
d
d
e
e
NT
#2
#6
#1
#4
#5
#3
#9
#10
#7
PvARL1-RNAi
A
B
a
b
g
g
Supplemental Figure S1. Relative expression of PvARL1 in PvARL1-OE and PvARL1-RNAi transgenic switchgrass.
A, Relative expression of PvARL1 in PvARL1-OE; B, Relative expression of PvARL1 in PvARL1-RNAi. Asterisks represent significant differences at P < 0.05.

## Slide 2
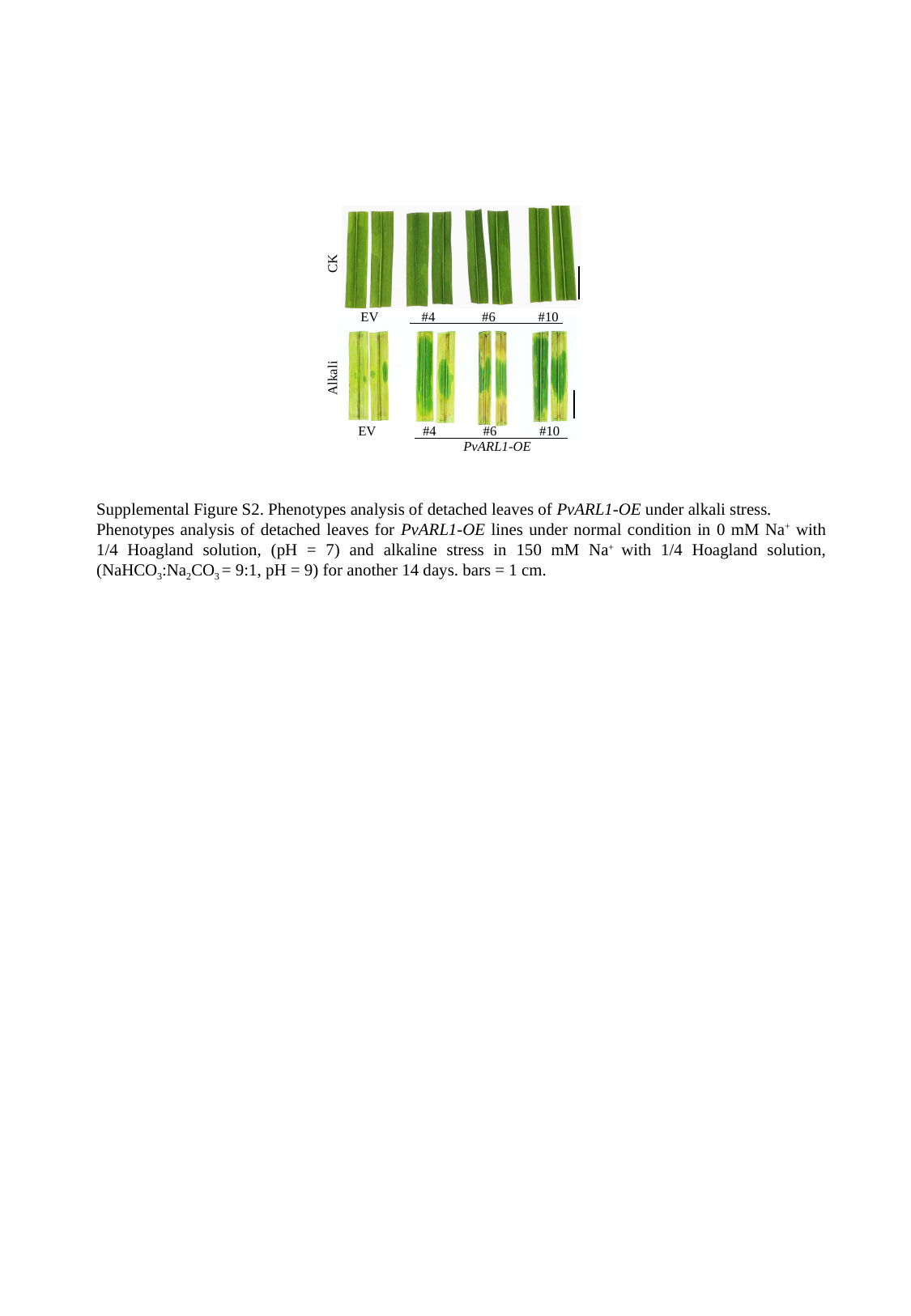

EV
#4
#6
#10
EV
#4
#6
#10
PvARL1-OE
CK
Alkali
Supplemental Figure S2. Phenotypes analysis of detached leaves of PvARL1-OE under alkali stress.
Phenotypes analysis of detached leaves for PvARL1-OE lines under normal condition in 0 mM Na+ with 1/4 Hoagland solution, (pH = 7) and alkaline stress in 150 mM Na+ with 1/4 Hoagland solution, (NaHCO3:Na2CO3 = 9:1, pH = 9) for another 14 days. bars = 1 cm.

## Slide 3
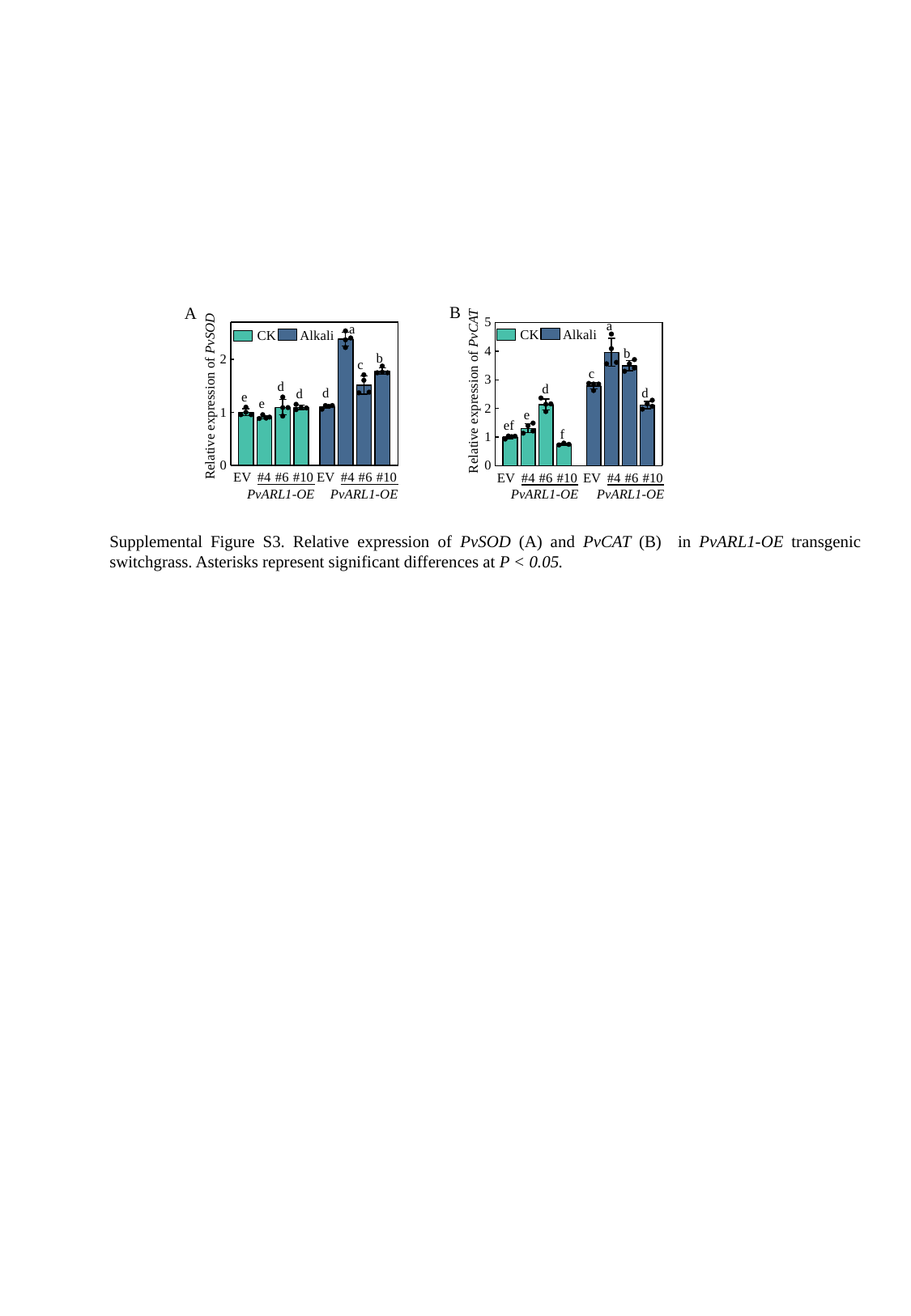

B
A
a
a
b
b
c
d
Relative expression of PvCAT
d
Relative expression of PvSOD
e
EV
#4
#6
#10
EV
#4
#6
#10
EV
#4
#6
#10
EV
#4
#6
#10
PvARL1-OE
PvARL1-OE
PvARL1-OE
PvARL1-OE
Alkali
CK
Alkali
CK
c
d
d
d
e
e
ef
f
Supplemental Figure S3. Relative expression of PvSOD (A) and PvCAT (B) in PvARL1-OE transgenic switchgrass. Asterisks represent significant differences at P < 0.05.

## Slide 4
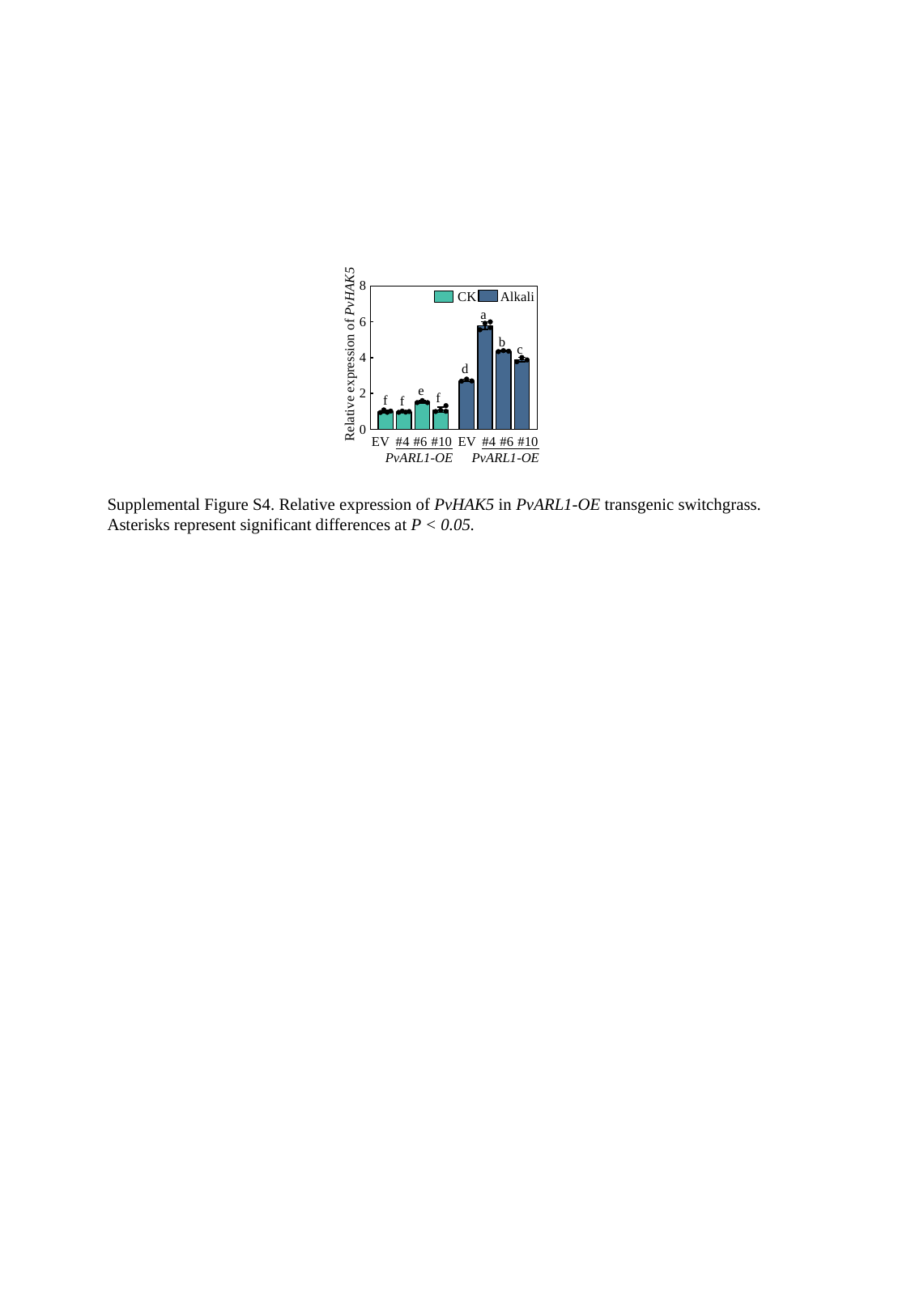

a
b
c
Relative expression of PvHAK5
e
EV
#4
#6
#10
EV
#4
#6
#10
PvARL1-OE
PvARL1-OE
Alkali
CK
d
f
f
f
Supplemental Figure S4. Relative expression of PvHAK5 in PvARL1-OE transgenic switchgrass.
Asterisks represent significant differences at P < 0.05.
